# The Sickle Effect: The Silent Titan Affecting Glycated Hemoglobin Reliability

**DOI:** 10.7759/cureus.9685

**Published:** 2020-08-12

**Authors:** Domonick K Gordon, Madiha Hussain, Prabhat Kumar, Sara Khan, Safeera Khan

**Affiliations:** 1 Internal Medicine, California Institute of Behavioral Neurosciences & Psychology, Fairfield, USA; 2 Internal Medicine, Scarborough General Hospital, Scarborough, TTO; 3 Neuropsychiatry, California Institute of Behavioral Neurosciences & Psychology, Fairfield, USA; 4 Gastroenterology and Hepatology, Cleveland Clinic Foundation, Cleveland, USA; 5 Medicine and Surgery, Bangalore Medical College & Research Institute, Bangalore, IND; 6 Medicine, California Institute of Behavioral Neurosciences & Psychology, Fairfield, USA

**Keywords:** sickle cell trait, hbas, hba1c, glycosylated hemoglobin, diabetes, rbc lifespan, race, genetics

## Abstract

Hemoglobin A1c (HbA1c) is a popular invaluable tool in the diagnosis of Type 2 diabetes for red blood cells (RBCs) with a lifespan of 120 days; however, many factors, including hemoglobinopathies, affect its accuracy. Sickle cell trait, primarily a benign medical condition, is a point mutation in only one of two beta-globin genes on chromosome 11. We performed a traditional review to identify how the sickle cell trait (SCT) affects the interpretation of HbA1c and the further implications it may have on the diagnosis and management of Type 2 diabetes.

A literature search was performed using PubMed®/MEDLINE® and Google Scholar with formulated keywords (sickle cell trait, HbAS, HbA1c, glycosylated hemoglobin, diabetes, RBC lifespan, race, and genetics), with the majority of results being mainly observational studies. The National Glycohemoglobin Standardization Program (NGSP) is responsible for standardizing HbA1c results and also highlights factors that can interfere with HbA1c, including hemoglobin variants. Studies that utilize only an NGSP-certified method with no clinically significant interference by HbS in patients with and without SCT showed contrasting results. Additional studies showed that persons of African ancestry, the group to which the majority of SCT patients belong, have a higher HbA1c than non-Hispanic whites (NHWs), just based on race, and a greater probability of having glucose-6-phosphate dehydrogenase (G6PD) deficiency, which lowers HbA1c. The most extensive study investigating the RBC lifespan in SCT patients showed a reduction in the cell lifespan compared to normal patients; however, other smaller studies were contradictory.

Our study highlights the need for hemoglobinopathy detection before or during HbA1c measurement in populations with a high degree of African ancestry and the importance of patient notification. It also shows that SCT affects the accuracy of HbA1c, through its likely reduction of RBC lifespan and its increased association with African ancestry and G6PD deficiency. This review recommends that for SCT patients with potential Type 2 diabetes, HbA1c should be used in combination with another diagnostic tool such as fasting blood glucose, fructosamine, or glycated albumin to decrease the chances of a missed diagnosis.

## Introduction and background

The year 2020 marks the 110th anniversary of the medical discovery of sickle cell disease (SCD), the first inherited disease ever identified at the molecular level though present for thousands of years prior. Sickle cell disease, called so due to red blood cells' (RBCs) similar appearance to a simple agricultural tool in this, now affects millions of people worldwide, mainly in areas where malaria was or still is present, as well as through international migration.

**Figure 1:**
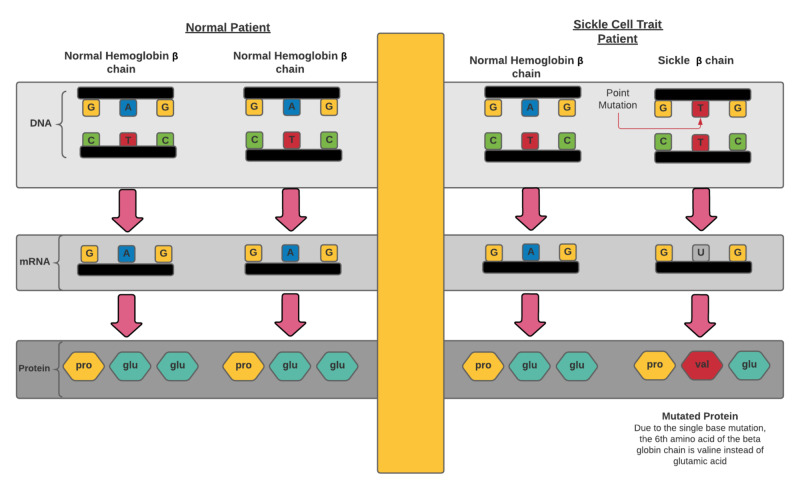
Comparison of β-globin chains of hematologically normal and sickle cell trait patients

Sickle cell trait (SCT), which offers protection against malaria, is a point mutation in only one of two beta-globin genes on chromosome 11, as seen in Figure 1, which differentiates it genetically from SCD. SCD (genotype hemoglobin [Hb] SS, HbSC, and other variants) clinically presents with a range of complications, while SCT (genotype HbAS) is mainly considered a benign condition; however, complications such as venous thromboembolism, exercise-related injury, and renal complications have been identified [1].

The UK Prospective Diabetes Study (UKPDS) and Diabetes Control and Complications Trial (DCCT) [2] have established that there is a clear relationship between mean blood glucose and vascular complications. National Glycohemoglobin Standardization Program (NGSP) is responsible for standardizing HbA1C results, so that laboratory results are comparable to those reported in DCCT. It is known that SCT affects some methods used in HbA1c interpretation. The main methods of measuring HbA1C include capillary electrophoresis, boronate affinity high-performance liquid chromatography (HPLC), ion-exchange HPLC, immunoassay, and enzymatic assays. Multiple types of research have shown that SCT does not affect HbA1C [3], leads to higher [4], or lower HbA1C compared to those without SCT [5]. To address the analytical aspect to some extent, the NGSP has a table, as shown in Table 1, which highlights the interference of HbS in the most frequently used methods for the evaluation of HbA1C.

**Table 1:** Most typical methods used in measuring HbA1c and the effect of the HbS variant Hb, hemoglobin; HPLC, high-performance liquid chromatography

Does the HbS variant affect the HbA1c method?		
No	Yes	
	Increased (↑ HbA1c)	Decreased (↓ HbA1c)
Abbott Architect c Enzymatic	Beckman AU system (reagent lot OSR6192, lot B00389 not yet evaluated)	Tosoh G8 (not including version 5.24)
Alere Afinion	Polymer Tech Systems A1cNOW	
Arkray ADAMS A1c HA-8180V (Menarini)		
Beckman Synchron System		
Beckman HbA1c Advanced B93009 Online Application on DxC 700 AU		
Bio-Rad D-10 (A1c program)		
Bio-Rad D-100 (A1c program)		
Bio-Rad Variant II NU		
Bio-Rad Variant II Turbo		
Bio-Rad Variant II Turbo 2.0		
Ortho-Clinical Vitros		
Roche Cobas Integra Gen.2		
Roche/Hitachi (Tina Quant II)		
Sebia Capillarys 2 Flex Piercing		
Siemens Advia A1c (new version)		
Siemens DCA 2000/Vantage		
Siemens Dimension		
Tosoh G7		
Trinity (Primus) HPLC (affinity)		

However, a recent study by Lacy et al. showed that using an NGSP-certified method with no clinically significant interference by HbS in African Americans with and without SCT still showed significantly lower HbA1C in those with SCT [6]. This unexpected result led to the questioning of what other factors in the SCT group affect the interpretation of HbA1C other than the analytical method used.

As reported by the International Diabetes Federation, the prevalence of Type 2 diabetes mellitus (T2DM) in 2019 was approximately 463 million (20-79 years), with an estimated projection of 700 million in the year 2045. The estimated number of SCT patients worldwide is approximately 100 million [7]. These epidemiological statistics suggest an increased chance and a likely growing number of patients with concomitant T2DM and SCT. HbA1C is a measure of glycation of hemoglobin in RBCs. It is a popular invaluable tool used in screening, diagnosis of T2DM, and assessment of glucose control over an 8- to 12-week period for RBCs with a lifespan of 120 days [8,9].

SCT being a majorly benign medical condition is likely underdiagnosed due to lack of clinical sequelae and, therefore, could be present in numerous patients unknowingly. It is still unknown whether the RBC lifespan in SCT patients is the same as a hematologically normal patient. RBC survival is a determinant of HbA1C concentration [10]; if this is hypothetically different in SCT patients, how does this affect the relationship between average blood glucose and HbA1C? What factors other than analytical, such as race, genetics, erythrocyte survival, affect the interpretation of HbA1C in SCT patients? If SCT underestimates HbA1c, then this leads to a decrease in the number of SCT patients diagnosed with T2DM, late diagnosis of T2DM with an increased risk of complications, and also gives those known SCT patients with T2DM a false sense of security. If SCT overestimates HbA1c, this may lead to more aggressive treatment of diabetes, increasing the risk of hypoglycemia.

We intend to conduct a traditional review to identify how SCT affects the interpretation of HbA1C and the further implications it may have in the diagnosis and management of T2DM.

## Review

Methods

Literature Search

We performed a literature search using PubMed®/MEDLINE® and Google Scholar using five formulated keywords, as seen in Table 2.

**Table 2:** Electronic search results showing the number of articles resulting from each of the determined keywords Hb, hemoglobin; RBC, red blood cell

Keyword used	Date	Database searched	No. of papers/results
Sickle cell trait and RBC lifespan	June 2020	Google Scholar	6160
Sickle cell trait and HbA1c	June 2020	Google Scholar	675
Sickle cell trait and diabetes	June 2020	PubMed	130
Race and HbA1C	June 2020	PubMed	2791
Genetics and HbA1c	June 2020	PubMed	3362

Each article's abstract was individually read and used to determine its relevance. Once deemed relevant, the article was obtained via free access through the search engine, a librarian, or online payment for the article through the respective journal. We reviewed each relevant article, and their reference list to supplement our search for articles not captured by the database searches.

Selection Criteria

This review included only articles published in the English language, involving adult human patients. We included all study types and study designs from all geographic locations. Quality assessment tools were not performed. The selection criteria were slightly different, based on the keyword used:

i. "Sickle cell trait and RBC lifespan"

We identified only a small number of relevant articles, and as such, no filter was applied.

ii. "Sickle cell trait and HbA1c" and "sickle cell trait and diabetes"

Only articles over the past 20 years were included. Articles that focused on how the different methodologies affect HbA1C in SCT patients were excluded, as this information is already available, as seen in Table 1. Studies that did not use an NGSP-certified method with minimal HbS effect, and those that did not mention the methodology used were also excluded from this study.

iii. "Race and HbA1c"

Only articles over the past 20 years were included.

iv. "Genetics and HbA1c"

Only articles over the past five years were included.

Results

After modifying the database searches with the inclusion/exclusion criteria, 11 articles were identified that utilized an NGSP-certified method with no clinically significant interference by HbS. Table 3 compares the results of all 11 studies obtained among participants with and without SCT.

**Table 3:** Comparison of studies utilizing an NGSP-certified method with no clinically significant interference by HbS in participants with and without SCT NGSP, National Glycohemoglobin Standardization Program; Hb, hemoglobin; PCA, principal component analysis; FG, fasting glucose; PEA, percent of European ancestry; GWAS, genome-wide association studies; NHWs, non-Hispanic Whites; T2DM, Type 2 diabetes mellitus; OGTT, oral glucose tolerance test; SCD, sickle cell disease; SCT, sickle cell trait; YOP, year of publication; SE, standard error

Author and YOP	Purpose of the study	Total no. of participants	Study type	Results	Conclusion
					HbA1c in SCT patients vs. hematologically normal patients
Hivert et al. (2018) [4]	Three genetic markers were used to explain HbA1c differences between NHWs and Black patients. The three parameters were (i) genetic variants known to cause hemoglobinopathies, (ii) associated with HbA1c discovered in the GWAS meta-analysis, and (iii) PCA factors capturing continental ancestry derived from genetic variants distributed across the genome	2658	Observational study (clinical research article)	In Black patients, the genetic variant causing SCT (rs334) was associated with higher HbA1c (β [SE] = +0.44 [0.08]%; P=2.1 x 10^-4^) despite adjustments made for fasting glucose, BMI, waist circumference and PCA factors	Higher
Skinner et al. (2019) [5]	Comparison between HbA1c, FG, and fructosamine in patients with and without SCT (with normal glycemic control and T2DM) living in Senegal	203	Observational study (case-control)	HbA1c was able to classify 28% of SCT group patients with hyperglycemia vs. 40% and 45% by FG and fructosamine	Lower
Echouffo-Tcheugui et al. (2019) [11]	Black patients without diabetes were investigated to determine whether the proportion of European genetic ancestry (PEA) mediates the SCT and HbA1c relation	3569	Observational study	HbA1c was significantly lower in the SCT group vs. the non-SCT group (difference 0.23 [SE 0.04], P<0.001). The association between SCT and HbA1c was β_SCT _[SE] = -0.18 [0.03], P<0.001	Lower
Briker et al. (2019) [12]	In Africa-born Black patients living in America, they aimed (1) to determine the nutritional and hematologic profiles, (2) to determine glucose tolerance categorization by HbA1c and OGTT, (3) to determine the diagnostic reproducibility of OGTT and HbA1c and (4) In T2DM and prediabetes, to compare the degree of glycemia, insulin resistance and beta-cell function detected by HbA1c vs. that detected by OGTT and not HbA1c	430	Observational study (cross-sectional)	HbA1c in HbAA T2DM = 6.7 ± 1.7; HbA1c in HbAS and HbAC T2DM = 5.7 ± 0.9, P=0.120; HbA1c in HbAA prediabetic patients = 5.6 ± 0.4; HbA1c in HbAS and HbAC prediabetic patients = 5.6 ± 0.5, P=0.581	Same
Adroja et al. (2018) [13]	To analyze various hemoglobin subfractions in patients with SCD and SCT and study variation in HbA1c when compared to blood glucose levels	50	Prospective observational study	The highly variable and non-consistent values of glycosylated Hb under- or overestimates the glycemic control in patients with sickle cell disease and trait	Higher to lower
Raffield et al. (2018) [14]	To identify the association between common African ancestral mutations including, α- and β-thalassemia traits on clinical phenotypes	2916	Observational study (cohort)	SCT was associated with lower HbA1c	Lower
Lacy et al. (2017) [6]	(1) To examine the link between HbA1c and SCT while controlling for other measures of glucose levels, (2) to compare the prevalence of diabetes and prediabetes by SCT status, and (3) to determine if SCT alters the discriminative ability of HbA1c to classify individuals with prediabetes or diabetes	7938	Observational study (retrospective cohort)	Compared with participants without SCT, SCT had lower levels of HbA1c at any given concentration of fasting or 2-hr glucose	Lower
Wu et al. (2018) [15]	To evaluate the HbA1c-glucose association between African Americans with and without SCT, and Whites using data from two cohort studies	6623	Observational (cohort)	African Americans with SCT have at least the same to lower mean HbA1c values than Whites despite higher glycemia levels	Same to lower
Sumner et al. (2015) [3]	To determine the diagnostic ability of A1C in Africans with heterozygous hemoglobinopathies	216	Observational study	Using A1C and variant hemoglobin into the model, the 2-hr glucose test was not significant (OR 1.07 [95% CI 0.52, 2.18]); A1C sensitivities for the normal and variant hemoglobin groups were 54% vs. 47% (P=0.59)	Same
Bleyer et al. (2010) [16]	To determine the effect of SCT on the measurement of glycated hemoglobin (HbA1c) in African American patients with diabetes mellitus	885	Observational (retrospective study)	HbA1c (%) in African Americans with no trait 7.2 ± 1.1; HbA1c (%) in African Americans with SCT was 7.4 ± 1.1	Same
Camargo et al. (2004) [17]	To identify the causes of very low glycohemoglobin values in patients with diabetes in Southern Brazil	29,657	Observational study	One hundred thirty patients were identified with an HbA1C ≤ 4.7, with the majority of patients with a Hb variant being SCT	Lower

The database searches also identified six articles that attempt to determine the RBC lifespan in SCT patients, and the results of all these studies are compared in Table 4.

**Table 4:** Comparison of studies attempting to determine the RBC lifespan in SCT patients YOP, year of publication; SCT, sickle cell trait; SCD, sickle cell disease; RBC, red blood cell; MCL, mean cell lifespan; ^51^Cr, radioactive sodium chromate; ^32^DFP, radioactive disisopropylflurophosphate; T½, half-life

Author and YOP	Purpose of the study	Methodology	No. of participants		Study type	Limitations	Results		Conclusion
			Total	SCT patients			Normal patients	SCT patients	
Barbedo et al. (1974) [18]	To determine RBC lifespan in SCT patients comparing ^51^Cr and ^32^DFP	^51^Cr and ^32^DFP	5	5	Observational study	Two included patients had pernicious anemia and slightly reduced iron levels	T½ of ^51^Cr > 26 days; MCL with ^32^DFP was 100-125 days	T½ of ^51^Cr 28.5-32.1 days (30.3 ± 1.8 days); MCL with ^32^DFP was 95.1-119.7 days (107.4 ± 12.3 days)	Normal; note well that one SCT patient had a decreased ^32^DFP of 95.7 days, but this was attributed to a possible experimental error
McCurdy et al. (1969) [19]	To compare the red cell survival curves obtained using the chromium tag with those using ^32^DFP in patients with abnormal hemoglobin	^51^Cr and ^32^DFP	28	4	Observational study	The patients categorized as normal were not completely free from disease	T½ of ^51^Cr 12.6-27.7 days; MCL with ^32^DFP was 21.4-102.5 days	T½ of ^51^ Cr was 24.3-30.1 days; MCL with ^32^DFP was 91.2-92.4 days; note well that one SCT patient had α-thalassemia and the other three had associated elliptocytosis	Normal
Suarez et al. (1959) [20]	To determine the incidence of hemoglobinopathies in different racial groups in Puerto Rico and the relationship between the rate of destruction of red cells to the identified hemoglobinopathy in the population	^51^Cr	2089	30	Observational study	There was no correction for elution of ^51^Cr from the RBCs	T½ of ^51^Cr is 24.5 days	T½ of ^51^Cr is 20.6 days	Decreased
Weinstein et al. (1954) [21]	To determine RBC survival in patients with hemoglobinopathies using ^51^Cr	^51^Cr	12	2	Observational study	There was no correction for elution of ^51^Cr from the RBCs	T½ of ^51^Cr is 29.9-36.3 days (33.1 ± 3.2 days)	Mean T½ of ^51^Cr is 32.5 days (27 and 38 days for the two patients)	Normal
Callender et al. (1949) [22]	Determination of the survival times of SCT and SCD erythrocytes post-transfusion	Ashby technic	41	6	Observational study	Lack of hemoglobin proportions of the SCT patients	Normal survival of blood donated from hematologically normal patients transfused into hematologically normal patients, patients with hypochromic anemia and sickle cell disease patients	Normal survival of blood donated from SCT patients transfused into hematologically normal patients (in four out of five transfusions), SCD patients, and a patient with Eisenmenger complex	Normal
Singer et al. (1948) [23]	Determinations of the survival times of sickle cells	Ashby technic	13	6	Observational study	Lack of hemoglobin proportions of the SCT patients	The normal survival of hematologically normal transfused RBC is 120 days	When SCT cells are transfused into an SCD patient, the transfused cells survive 120 days	Normal

Discussion

Comparison of Studies Using an NGSP-Certified Method With No HbS Interference in SCT Patients

The American Diabetes Association (ADA) recommends that patients with SCT have HbA1c measurements using an NGSP-certified device without HbS interference. With this acknowledgment, it would be understandable to assume that using an NGSP-certified device without HbS interference in a SCT patient should give reliably similar HbA1c results for estimated average glucose over the previous 120 days compared to a hematologically normal patient.

However, 11 studies showed that using an NGSP-certified machine without HbS interference gave conflicting results, as seen in Table 3. One study stated that HbA1c was higher [4], three stated the same [3,12,16], five stated lower [5,6,11,14,17], one stated higher to lower [13], and one stated the same to lower [15], when compared to a hematologically normal patient.

These studies showed some limitations: lack of checking for alpha thalassemia [5], low frequency of SCT patients [3,4,6,12], and basing the analysis on a single determination such as oral glucose tolerance test (OGTT) [4], fasting plasma glucose (FPG), or HbA1c [3]. Lack of HbS percentages [14] and exclusion of participants, who may have SCT from the analysis process [6], due to missing data of SCT status may contribute to the differences in results obtained.

HbA1c should have no significant difference among SCT patients compared to hematologically normal patients if the RBC lifespan in SCT is normal, as reported by the ADA. Accounting for most controllable factors, the unexpected contrasting findings of Table 3 suggest that there are other factors in SCT patients affecting HbA1c, or the RBC lifespan is not the same as that in a hematologically normal patient.

To find the non-analytical reasons for this difference, we have sought to review articles that address race, genetics, and RBC lifespan in SCT patients.

The Racial Effect on HbA1c: The Implications in SCT Patients

Since commencing the use of HbA1c in T2DM diagnosis in 2009, there has been a contention of how racial differences and nonglycemic factors may affect interpretation. It is well known and accepted that race affects HbA1c and that the HbS hemoglobinopathy occurs more frequently in Black persons than non-Hispanic whites (NHWs). A systematic review of 12 studies using data from 49,238 patients concluded that in patients without DM, HbA1c is higher in Blacks, Asians, and Latinos than White persons [24]. Numerous studies have supported this conclusion, including a meta-analysis of 391 non-diabetic participants, which concluded higher HbA1c among African Americans despite adjustments for plasma glucose and other characteristics that correlate to HbA1c [25].

The results are similar for studies using patients with impaired glucose tolerance (IGT). A retrospective cross-sectional study of 3548 participants showed higher HbA1c in Black versus White persons [26]. In another study including only patients with IGT, 3189 participants from five racial groups after adjusting for characteristics that correlate to HbA1c, racial disparity still existed. Post-adjustment mean HbA1c values were 6.18% for Blacks (P<0.001), 6.12% for American Indians, 6.00% for Asians, 5.93% for Hispanics, and 5.78% for NHWs [27]. The conclusion from this study is that among patients with IGT, HbA1c may not be a useful tool for evaluating and comparing glucose control across racial groups.

The reason for these observed differences between races is not known. Suggestions include differences in hemoglobin glycation, non-glycemic genetic determinants, RBC survival, and differences in extra- and intracellular glucose balance [10,28].

Though not widely recognized, important limitations of HbA1c in non-diabetic patients include diet, BMI, age, gender, and glycemia, which explained less than one-third of variance [29]. This variation in patients with normal glucose levels may be due to genetics affecting hemoglobin glycation, inter-individual red cell turnover, and differences in intra-erythrocyte/extra-erythrocyte environment [30-33]. Table 5 shows some other sources of error when using HbA1c to estimate glycemic control.

**Table 5:** Sources of error in estimating HbA1c Hb, hemoglobin; G6PD, glucose-6-phosphate dehydrogenase; NNRTI, non-nucleoside reverse transcriptase inhibitor; PI, protease inhibitor; SCD, sickle cell disease

Falsely low HbA1c	Falsely high HbA1c
Anemia	Chronic kidney disease
Chronic kidney disease	Folate deficiency
Increased RBC turnover, for example, erythropoietin therapy, SCD, G6PD deficiency, hemodialysis, hemolytic anemia, pregnancy (2nd and 3rd trimester), recent blood loss/heavy bleeding transfusion	Iron deficiency
Patients being treated for iron, B12, and folate deficiencies	Vitamin B12 deficiency
Recovery from acute blood loss	
Medications: antiretrovirals (e.g., NNRTIs), aspirin (small doses), dapsone, hydroxyurea, ribavirin, trimethoprim-sulfamethoxazole, vitamins C and E	Medications: antiretrovirals (e.g., PIs), aspirin (large doses), chronic opiate use

As most SCT patients are Black persons [34], and these individuals have a higher HbA1c compared to NHWs, this will likely be associated and/or contribute to the differences observed in Table 3. Until the identification of the reasons for the differences in race, HbA1c as the sole tool for the diagnosis of T2DM in Black patients, including Black SCT patients, may not be advisable, due to the potential risk of error and misclassification.

Genetics and HbA1c: The Implications in SCT Patients

To further understand the genetic factors responsible for the difference in HbA1c between the races, one of the most extensive genome-wide association studies (GWAS) conducted a meta-analysis of 159,940 persons from 82 cohorts. These cohorts included persons from South Asian, European, African, and East Asian ancestries and were able to identify 60 common genetic variants associated with HbA1c [35].

Of these 60 independent variants associated with HbA1c, there were 22 erythrocyte variants, affecting the structure, lifespan, and function of RBCs, and 19 glycemic variants influencing glucose control. These variants support the hypothesis of inter-individual intracellular and extracellular differences, which affect HbA1c. Results from the continued follow-up of 33,000 people from five ancestry groups showed that the higher the number of glycemic variants, the greater the risk of diabetes (odds ratio [OR] = 1.05 per HbA1c-raising allele, P=3 × 10^−29^). Contrastingly, the more erythrocytic variants that a person had did not increase the risk of diabetes, as some erythrocytic variants, such as glucose-6-phosphate dehydrogenase (G6PD), could lower HbA1c independent of glucose concentration and lead to a missed diagnosis.

Taking all the 60 variants (glycemic and erythrocytic) into account, for anyone who was not of African descent, patients who had more versus those who had fewer variants showed minimal difference in HbA1c (approximately 0.2 units). Of those with African ancestry, however, patients who had more versus those who had fewer variants showed a massive difference in HbA1c (0.8 units), mainly due to the erythrocytic variant G6PD found. Of note, almost no one from any ancestry carries this variant, except people of African ancestry, where up to 11% carry at least one copy of this variant.

G6PD deficiency is an X-linked recessive disorder and is the most common enzyme deficiency worldwide. G6PD, like SCT, offers some protection from malaria, and most patients are asymptomatic. Factors that evoke oxidant stress, such as fava beans and numerous drugs (e.g., antimalarials), can result in acute hemolytic anemia in these patients. The global prevalence of G6PD is approximately 4.9%, representing 330 million people, with African countries generally having the highest prevalence. People of African ancestry are at a higher risk of G6PD deficiency [36].

Briker et al. also found similar disparities in the African population [12]. Using HbA1c as the sole diagnostic tool in nutritionally replete Africans without anemia or heterozygous hemoglobinopathy would underdiagnose 60% of diabetics and 40% of prediabetics.

Another possible reason for racial differences in HbA1c is the glycation gap, which is a measure of the difference between glycation of extracellular plasma protein (fructosamine or glycated albumin) and intracellular hemoglobin (HbA1c). The glycemic gap is reproducible for individuals [37,38], reflecting the correlation between extracellular and intracellular glycation. Also, the high hereditability among identical non-diabetic twins suggests that there is likely a genetic link to the glycation gap [33].

**Figure 2:**
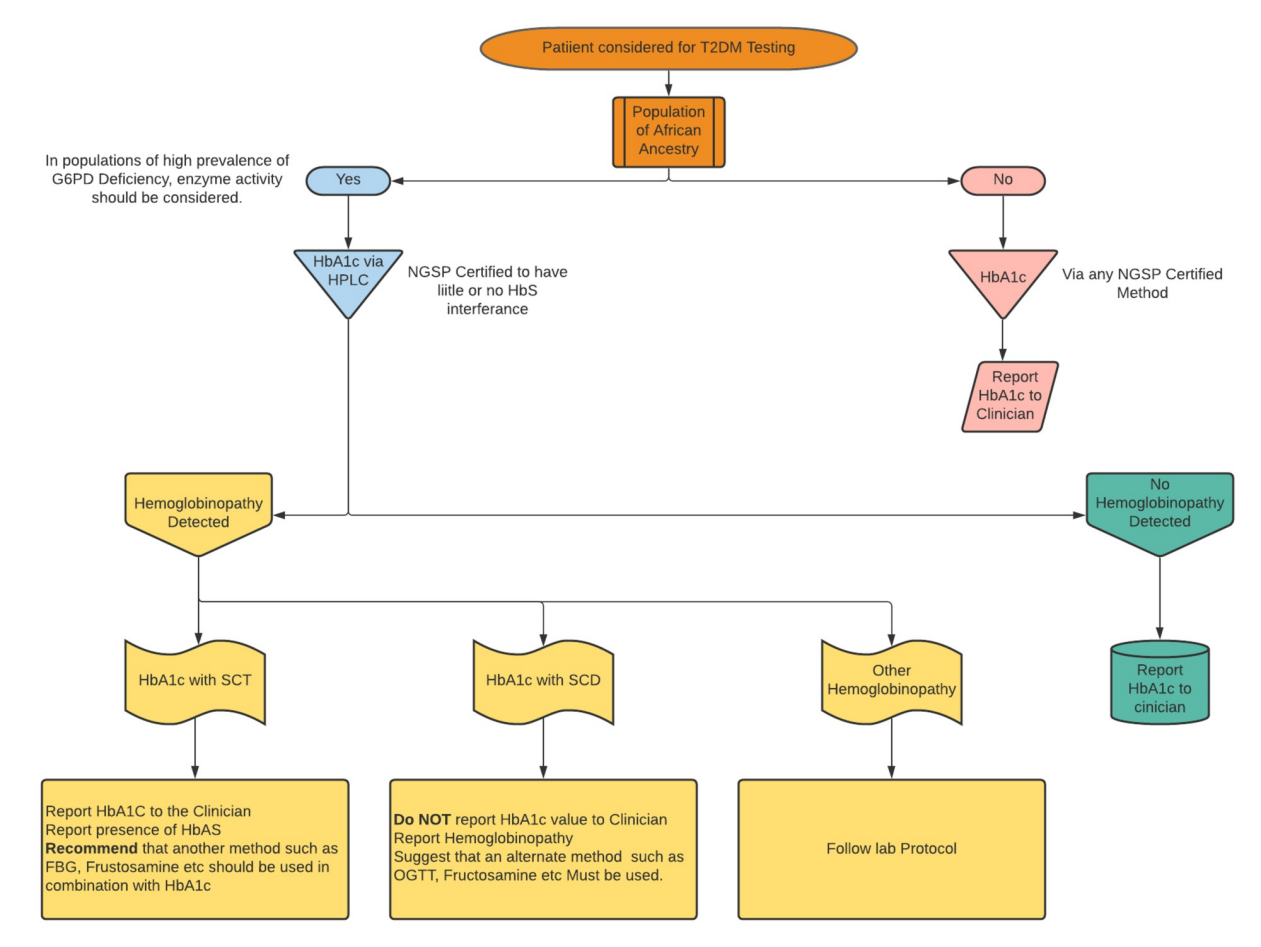
Use of HPLC for T2DM screening in populations with significant HbS variants HPLC, high-performance liquid chromatography; Hb, hemoglobin; G6PD, glucose-6-phosphate dehydrogenase deficiency; NGSP, National Glycohemoglobin Standardization Program; SCD, sickle cell disease; SCT, sickle cell trait; T2DM, Type 2 diabetes mellitus; OGTT, oral glucose tolerance test; FBG, fasting blood glucose

Figure 2 suggests a protocol for the use of HbA1c in populations where there is a high African ancestry, which also positively correlates to populations of high HbS hemoglobinopathy. This suggested protocol is proactive, finding hemoglobinopathy at the onset of determining if the patient has diabetes, instead of responding to conflicting tests results. Hemoglobin electrophoresis may be an alternative to HPLC for hemoglobinopathy detection. It is crucial that once the lab detects abnormal hemoglobin, this information should accompany the HbA1c results with suggestions regardless if the variant does not affect the methodology. The diagnosis of SCT though primarily silent can have some clinical sequelae [1]. The knowledge of their genetic status can allow the patients to inform other family members to get tested, especially in countries with a high HbS prevalence with no neonatal screening.

With this information, populations with a significant African ancestry should consider having genetic testing for G6PD before HbA1c testing. As most SCT patients are of African ancestry, there is likely a subset of SCT patients with associated G6PD deficiency. HbA1c as the sole tool for diagnosing T2DM in Black patients, including Black SCT patients, where the survival of SCT RBCs is questionable may lead to erroneous results.

Pathophysiology of SCT: How Does RBC Lifespan in SCT Affect HbA1c?

**Figure 3:**
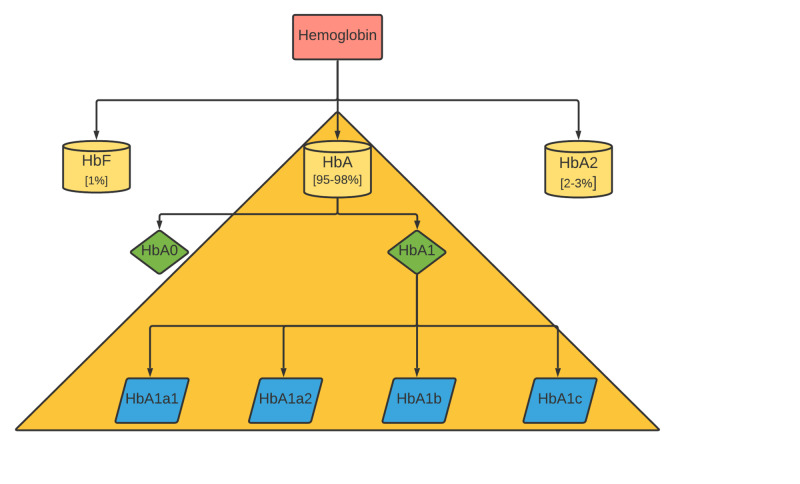
Major subtypes of adult hemoglobin

The main types of hemoglobin in adults include fetal hemoglobin (HbF α2γ2) (1%), hemoglobin A2 (HbA2 α2δ2) (2%-3%), and hemoglobin A (HbA α2β2) (95%-98%) [39]. Subtypes of HbA detected by electrophoresis are HbA0, HbA1a1, HbA1a2, HbA1b, and HbA1c; Figure 3 shows the major subtypes of adult hemoglobin.

HbA1c represents the majority (70%-90%) of HbA1 and is the result of nonenzymatic glycosylation of the N- terminal of the β-globin chain in the presence of free sugars.

The average expected RBC lifespan is 120 days, with HbA1c forming gradually and continuously throughout its lifespan. HbA1c depends on a few factors: a constant erythrocyte lifespan, free permeability to glucose, and nonenzymatic glycation directly proportional to the glucose concentration [10]. Once these conditions are satisfied, the average blood glucose levels over the previous 120 days are represented by HbA1c in the hematologically normal patient and are useful in the diagnosis of diabetes [10].

The average lifespan of RBCs in HbAS patients is widely accepted as normal without a highlighted specific reference [40]. We hypothesize that the lifespan of RBCs in SCT patients is reduced due to hemolysis, and therefore, there is less available time for the glycation of hemoglobin, which in turn may affect HbA1c. There, however, is limited data available to support this hypothesis. In one study, the RBC survival was 93 days [19], approximately 20% less than that of an average normal RBC. This reduction is assumed not to affect the rate of glycosylation and, therefore, HbA1c; however, is this irrefutably true?

Six identified studies have tried to determine if SCT patients have normal, reduced, or increased RBC survival compared to hematologically normal patients. These studies used three methods, namely, Ashby technic, radioactive sodium chromate (^51^Cr), and radioactive disisopropylflurophosphate (^32^DFP), as seen in Table 4. The two earlier studies using Ashby technic showed normal results [22,23]. In one, using ^51^Cr, two SCT patients had normal results [21], while in another study [20], 30 patients with SCT showed a reduced average half-life of RBCs from 24.5 days to 20.6 days.

The two most recent studies assessing RBC survival in SCT patients used ^51^Cr and ^32^DFP in four and five SCT patients, respectively, with both results showing normal survival [18,19]. The last study [18], but none of the previous five studies, mentioned family studies or the SCT patients' hemoglobin proportions. With the lack of hemoglobin proportions, there is a possibility that some of the included SCT participants in the earlier studies were S-β thalassemia patients. None of the six studies mentioned the β-globin S haplotype of the included SCT patients. There are multiple β-globin S haplotypes representing the geographic region or ethnic group from which these patients inherited this mutation. These include Arab-Indian, Bantu, or Central African Republic, Benin, Cameroon, and Senegal [41]. It is not known if different haplotypes have different RBC lifespans or affect HbA1c differently.

^51^Cr binds to the β-chain [42], which may be altered in the β-S chain compared to the normal β-chain. The rate of ^51^Cr elution in patients with abnormal β-chains was 1.2% daily (range: 0.1%-2.7%, specifically 1%-1.4% for SCT patients with elliptocytosis) while that of RBCs with normal β-chains was 1.3% (range: 0.7%-1.6%), which seems similar [19]. The lifespan of ^51^Cr may be affected by different elution rates, differential labeling based on cell age, and variable labeling of different hemoglobins [43]. The average elution rate of 1.3% in "normal patients" included participants with underlying medical conditions, including hereditary spherocytosis and elliptocytosis, which are known to have abnormal red cells and shortened red cell lifespan (RCL). The inclusion of such participants increases the possibility of interference and erroneous results. The mean cell lifespan using ^32^DFP for SCT patients with elliptocytosis is 92.6 days (range 91.2-94.2 days), while that of included normal patients was 72.7 days (range 21.4-102.5), which may be due to the small number of patients and interference from concomitant medical conditions [19].

These studies have a relatively small number of SCT participants, and the study with the highest number of SCT patients (more than the other five studies combined) showed a decrease in RBC survival, which we are more inclined to consider based on the quantity of SCT patients [20]. The studies' discrepancies are possibly due to methodology, an insufficient number of SCT participants, the inclusion of S-β thalassemia patients, and possible interference from concomitant diseases.

We recommend that a study be performed with a large sample size of SCT patients confirmed with HbEP percentages to improve the study's power. The SCT patients should include patients from each of the known haplotypes, and there should be an exclusion of confounders such as alpha thalassemia, S-β thalassemia, G6PD deficiency, and any underlying medical condition that can affect RBC lifespan. There should be a control group, including hematologically normal patients comparable in age, gender, ethnicity, and exclusion criteria, as described before to accommodate or highlight any discrepancy in the methodology used. We recommend any method, including RBC tagging, which has been shown to have no interference with HbS and does not utilize the β-globin chain.

The extent to which the RBC lifespan reduction affects HbA1c is not known. Using RCL as a parameter, one study used a kinetic mass balance model with boundaries fitted according to the limits of the standard relationship for HbA1c and average glucose (AG) to predict the link between HbA1c and AG for altered RCL [44]. With a normal basis curve using an RCL of 120 days, the ratio AGRCL/AG120 is represented by the formula: AGRCL/(AG120) = 120/RCL. As mentioned earlier, the average RBC lifespan in SCT patients is approximately 93 days [19]. For ease and simplicity of calculation, we will assume the average SCT RBC lifespan is 90 days. Using an RBC lifespan of 90 days, the average glucose is represented by AG90 = 4/3 x (AG120). Therefore, for any HbA1c level received for a hematologically normal patient with an RCL of 120 days, a similar HbA1c level in an SCT patient with an RCL of 90 days would represent a higher average glucose level, approximately 4/3 that of the hematologically normal patient.

The ADA criteria for the diagnosis of diabetes include the following [40]:

i. Fasting blood glucose (FBG) ≥ 126 mg/dL (7.0 mmol/L)

ii. 2-hr plasma glucose ≥ 200 mg/dL (11.1 mmol/L) during a 75 g OGTT

iii. HbA1c ≥ 6.5% (48 mmol/mol)

iv. RBG ≥ 200 mg/dL (11.1 mmol/L) with classic symptoms of hyperglycemia or hyperglycemic crises.

This study showed a second way of interpreting the results of HbA1c in patients with altered RCL. It calculated the operative AG (oAG) for altered RCL and calculated HbA1c if that oAG was present in a patient with a normal RCL of 120 days. The results showed that for any given %HbA1c, a decrease in RCL of 90 days would require a proportional increase in the oAG, representing a higher HbA1c in a patient with a normal RCL of 120 days. For example, results obtained from the study showed that an oAG for a measured %HbA1c (NGSP) of 6% for an RCL of 90 days would be equivalent to %HbA1c (NGSP) of 7.5% for a normal RCL of 120 days. Therefore, an SCT patient with an RCL of 90 days who presents to a clinician with an HbA1c of 6% would not be diagnosed as having diabetes. However, his/her average glucose would be equivalent to an uncontrolled diabetic patient if his/her RCL was 120 days. This discrepancy shows that HbA1c can underdiagnose T2DM in SCT patients, putting them at higher risk of complications associated with T2DM. 

Biomarkers such as fasting glucose, fructosamine, and HbA1c are not interchangeable and can result in a missed diagnosis and inappropriate follow-up of T2DM, especially in SCT patients [5]. To overcome this limitation of HbA1c, we recommend that HbA1c be used in combination with another form of confirmatory testing in SCT patients being investigated for T2DM. The use of HbA1c with FPG in Black patients is superior to using either test alone [3]. Fructosamine and glycated albumin that are independent of erythrocytes are also possible alternatives.

Study Limitations

As we reviewed only articles published in the English language, there likely was the exclusion of valuable findings from articles published in other languages. The use of quality assessment tools for the included articles could have strengthened our article. Articles reviewed were from the year 2000 onwards only, which excludes important conclusions from earlier publications.

## Conclusions

The effect of SCT on the routine diagnosis of T2DM using HbA1c was assessed. Clinicians must be aware of how the HbS variant affects methodologies used in HbA1c interpretation. In populations with a high prevalence of the HbS variant, there should be early identification of hemoglobinopathy in the subject and careful selection of the methodology used to determine HbA1c. Our study highlights the direct effect of SCT on the accuracy of HbA1c, through its likely reduction of RBC lifespan and indirectly through its increased association with African ancestry and G6PD deficiency. T2DM diagnosis in SCT patients is multifaceted, and the use of additional diagnostic tools such as fructosamine and glycated albumin may assist in the accurate diagnosis of patients. This article is important as it highlights a group of primarily clinically silent but biochemically highly active patients who may be underdiagnosed through their unknown hemoglobinopathy.

This article would benefit the millions of SCT patients worldwide, whether known or unknown, through early recognition of the hemoglobinopathy before or during HbA1c testing as well as offering optimal recognition of T2DM through additional tests, which would increase early diagnosis and treatment with improved prognosis. Due to small sample sizes and conflicting limited articles, the RBC lifespan in SCT patients remains unknown. We recommend a case-control study using a large sample size of SCT patients from each known haplotype with the exclusion of confounders. The method used should have no interference with the HbS variant and does not utilize the beta-globin chain.

## References

[1] Naik RP, Haywood C Jr (2015). Sickle cell trait diagnosis: clinical and social implications. Hematology Am Soc Hematol Educ Program.

[2] The Diabetes Prevention Program Research Group (1999). The Diabetes Prevention Program. Design and methods for a clinical trial in the prevention of type 2 diabetes. Diabetes Care.

[3] Sumner AE, Thoreson CK, O'Connor MY (2015). Detection of abnormal glucose tolerance in Africans is improved by combining A1C with fasting glucose: the Africans in America Study. Diabetes Care.

[4] Hivert MF, Christophi CA, Jablonski KA (2019). Genetic ancestry markers and difference in A1c between African American and White in the Diabetes Prevention Program. J Clin Endocrinol Metab.

[5] Skinner S, Diaw M, Ndour Mbaye M (2019). Evaluation of agreement between hemoglobin A1c, fasting glucose, and fructosamine in Senegalese individuals with and without sickle-cell trait. PLoS One.

[6] Lacy ME, Wellenius GA, Sumner AE (2017). Association of sickle cell trait with hemoglobin A1c in African Americans. JAMA.

[7] Goldsmith JC, Bonham VL, Joiner CH, Kato GJ, Noonan AS, Steinberg MH (2012). Framing the research agenda for sickle cell trait: building on the current understanding of clinical events and their potential implications. Am J Hematol.

[8] American Diabetes Association (2020). 2. Classification and diagnosis of diabetes: Standards of Medical Care in Diabetes - 2020. Diabetes Care.

[9] Goldstein DE, Little RR, Lorenz RA, Malone JI, Nathan D, Peterson CM, Sacks DB (2004). Tests of glycemia in diabetes. Diabetes Care.

[10] Herman WH, Cohen RM (2012). Racial and ethnic differences in the relationship between HbA1c and blood glucose: implications for the diagnosis of diabetes. J Clin Endocrinol Metab.

[11] Echouffo-Tcheugui JB, Mwasongwe SE, Sims M, Dagogo-Jack S, Golden SH, Correa A, Musani SK (2019). Sickle cell trait, European ancestry, and longitudinal tracking of HbA1c among African Americans: the Jackson Heart Study. Diabetes Care.

[12] Briker SM, Aduwo JY, Mugeni R (2019). A1C underperforms as a diagnostic test in Africans even in the absence of nutritional deficiencies, anemia and hemoglobinopathies: insight from the Africans in America study. Front Endocrinol (Lausanne).

[13] Adroja B (2020). Sumandeep Vidyapeeth Institutional Repository. Study of blood sugar levels and glycosylated hemoglobin in patients of sickle cell hemoglobinopathy (Doctoral dissertation). Sumandeep Vidyapeeth, Vadodara.

[14] Raffield LM, Ulirsch JC, Naik RP (2018). Common α-globin variants modify hematologic and other clinical phenotypes in sickle cell trait and disease. PLoS Genet.

[15] Wu WC, Lacy ME, Correa A, Carnethon M, Reiner AP, Eaton CB, Wellenius GA (2018). Association between hemoglobin A1c and glycemia in African Americans with and without sickle cell trait and Whites. Results from CARDIA and the Jackson Heart Study. J Diabetes Treat.

[16] Bleyer AJ, Vidya S, Sujata L (2010). The impact of sickle cell trait on glycated hemoglobin in diabetes mellitus. Diabet Med.

[17] Camargo JL, Gross JL (2004). Conditions associated with very low values of glycohemoglobin measured by an HPLC method. J Clin Pathol.

[18] Barbedo MM, McCurdy PR (1974). Red cell life span in sickle cell trait. Acta Haematol.

[19] McCurdy PR (1969). 32-DFP and 51-Cr for measurement of red cell life span in abnormal hemoglobin syndromes. Blood.

[20] Suarez RM, Buso R, Meyer LM, Olavarrieta ST (1959). Distribution of abnormal hemoglobins in Puerto Rico and survival studies of red blood cells using Cr51. Blood.

[21] Weinstein I, Spurling C, Klein H, Necheles TF (1954). Radioactive sodium chromate for the study of survival of red blood cells: III, the abnormal hemoglobin syndromes. Blood.

[22] Callender ST, Nickel JF (1949). Sickle cell disease: studied by measuring the survival of transfused red blood cells. J Lab Clin Med.

[23] Singer K, Robin S (1948). The life span of the sickle cell and the pathogenesis of sickle cell anemia. J Lab Clin Med.

[24] Cavagnolli G, Pimentel AL, Freitas PA, Gross JL, Camargo JL (2017). Effect of ethnicity on HbA1c levels in individuals without diabetes: systematic review and meta-analysis. PLoS One.

[25] Kirk J, Agostino R, Bell R, Passmore LV, Bonds DE, Karter AJ, Venkat Narayan KM (2006). Disparities in HbA1c levels between African-American and non-Hispanic White adults with diabetes: a meta-analysis. Diabetes Care.

[26] Ziemer DC, Kolm P, Weintraub WS (2010). Glucose-independent, black-white differences in hemoglobin A1c levels: a cross-sectional analysis of 2 studies. Ann Intern Med.

[27] Herman WH, Ma Y, Uwaifo G (2007). Differences in A1C by race and ethnicity among patients with impaired glucose tolerance in the Diabetes Prevention Program. Diabetes Care.

[28] Selvin E, Sacks DB (2017). Variability in the relationship of hemoglobin A1c and average glucose concentrations: how much does race matter?. Ann Intern Med.

[29] Yudkin JS, Forrest RD, Jackson CA, Ryle AJ, Davie S, Gould BJ (1990). Unexplained variability of glycated hemoglobin in non-diabetic subjects not related to glycemia. Diabetologia.

[30] Cohen RM, Franco RS, Khera PK (2008). Red cell life span heterogeneity in hematologically normal people is sufficient to alter HbA1c. Blood.

[31] Khera PK, Joiner CH, Carruthers A (2008). Evidence for inter-individual variation in the glucose gradient across the human RBC membrane and its relationship to Hb1c. Diabetes.

[32] Snieder H, Sawtell PA, Ross L, Walker J, Spector TD, Leslie RD (2001). HbA(1c) levels are genetically determined even in type 1 diabetes: evidence from healthy and diabetic twins. Diabetes.

[33] Cohen RM, Snieder H, Lindsell CJ (2006). Evidence for independent heritability of the glycation gap (glycosylation gap) fraction of HbA1c in non-diabetic twins. Diabetes Care.

[34] Ojodu J, Hulihan MM, Pope SN, Grant AM, Centers for Disease Control and Prevention (CDC) (2014). Incidence of sickle cell trait - United States, 2010. MMWR Morb Mortal Wkly Rep.

[35] Wheeler E, Leong A, Liu CT (2017). Impact of common genetic determinants of hemoglobin A1c on type 2 diabetes risk and diagnosis in ancestrally diverse populations: a transethnic genome-wide meta-analysis. PLoS Med.

[36] Nkhoma ET, Poole C, Vannappagari V, Hall SA, Beutler E (2009). The global prevalence of glucose-6-phosphate dehydrogenase deficiency: a systematic review and meta-analysis. Blood Cells Mol Dis.

[37] Cohen R, Holmes YR, Chenier TC, Joiner CH (2003). Discordance between HbA1c and fructosamine: evidence for a glycosylation gap and its relation to diabetic nephropathy. Diabetes Care.

[38] Nayak AU, Holland MR, Macdonald DR, Nevill A, Singh BM (2011). Evidence for consistency of the glycation gap in diabetes. Diabetes Care.

[39] Hanas R, John G, International HBA1c Consensus Committee (2010). 2010 Consensus statement on the worldwide standardization of the hemoglobin A1C measurement. Diabetes Care.

[40] American Diabetes Association (2020). Introduction: Standards of Medical Care in Diabetes - 2020. Diabetes Care.

[41] Loggetto SR (2013). Sickle cell anemia: clinical diversity and beta S-globin haplotypes. Rev Bras Hematol Hemoter.

[42] Pearson HA, Vertrees KM (1961). Site of binding of chromium-51 to hemoglobin. Nature.

[43] Pearson HA (1966). The binding of Cr51 to hemoglobin: II. In vivo elution rates of Cr51 from Hb CC, Hb CS and placental red cells. Blood.

[44] Molinaro R, Herman JH, Stickle DF (2017). Average glucose from hemoglobin A1c for altered red blood cell lifetimes: predictions based on a model for hemoglobin A1c formation. Clin Chim Acta.

